# 1D hydrogen engine modeling to investigate air-fuel ratio, spark timing, and water injection effects on performance and emissions

**DOI:** 10.1038/s41598-025-00952-0

**Published:** 2025-05-17

**Authors:** Omar Mohamed Saied, Mohamed Abdelwahab

**Affiliations:** 1 Automotive Mechatronics Department, Faculty of Engineering, German International University, Cairo, Egypt; 2 Automotive Department, Faculty of Engineering, Ain Shams University, 1 El Sarayat St. Abbasseya, El Weili, Cairo, 11535 Egypt

**Keywords:** Electrical and electronic engineering, Mechanical engineering

## Abstract

This study presents a comprehensive 1D modeling and simulation of a hydrogen-fueled internal combustion engine, focusing on the impacts of air-fuel ratio, spark timing, and water injection on performance and emissions. Using a single-cylinder BMW F650 GS engine as a baseline, simulations were conducted in Ricardo WAVE to evaluate engine behavior under varying conditions. Results demonstrate that hydrogen fueling reduces CO emissions to negligible levels but increases NO_x_ emissions under specific conditions due to elevated combustion temperatures. Water injection effectively mitigates NO_x_ formation and enhances volumetric efficiency while slightly compromising power output at high hydrogen-to-water ratios. The findings highlight hydrogen’s potential as a sustainable fuel for internal combustion engines and underline the importance of optimizing operating parameters to balance performance, efficiency, and emissions. This research contributes valuable insights into the development of cleaner, hydrogen-powered transportation solutions.

## Introduction

The global demand for energy is rapidly increasing due to factors such as population growth, industrial development, and urban expansion^1–3^. Projections estimate a 39% rise in global energy consumption over the next three decades. Currently, approximately 85% of the world’s energy comes from non-renewable sources, with fossil fuels constituting over 75% of the total energy supply^4–6^. However, fossil fuels have finite reserves and are concentrated in specific regions. Their continued use faces challenges from rising atmospheric CO_2_ levels, which have surpassed 36.57 billion tons, contributing to global warming and climate changes through the greenhouse effect^7–9^. Although the immediate halting of fossil fuel use presents difficulties, traffic-related emissions, which are responsible for over 70% of road emissions, significantly contribute to overall energy emissions^10^. Consequently, there is an increasing search for sustainable, low-carbon, or zero-carbon alternatives to mitigate emissions from ICEs^11–21^. Among these alternatives, hydrogen emerges as a particularly promising option for carbon-neutral energy in transportation and power applications^22^.

Building on this need for alternatives, the transport sector stands out as a major source of GHG emissions, contributing 16.2% of total global emissions in 2020, with 73.4% of these emissions originating from on-road transport^23^. As economic factors such as rising personal purchasing power and an increase in the number of light-duty vehicles drive an expected 20% increase in energy demand for transportation between 2019 and 2050^24^, it becomes imperative to explore various strategies for emission reduction. While enhancements in fuel conversion efficiency and the electrification of personal vehicle fleets could play a role in curbing this demand, commercial transportation is projected to expand significantly due to continued economic growth^25^. This context highlights the importance of decarbonizing heavy-duty vehicles, which has emerged as a central goal, particularly through the adoption of electrification and alternative propulsion systems^26^.

In light of these challenges, it is essential to consider the regulatory frameworks being established worldwide. Governments, including the European Union (EU) and the United States, have implemented stringent emissions reduction targets. The EU’s “Fit for 55” initiative aims for a 55% reduction in CO_2_ emissions from cars and vans by 2030, with an ambitious goal of achieving net-zero emissions by 2050. Similarly, the U.S. Environmental Protection Agency (EPA) has introduced rigorous multi-pollutant standards for light- and medium-duty vehicles for the period 2027–2032^27,28^. Although battery electric vehicles (BEVs) are a crucial part of meeting these regulatory standards due to their “zero emissions” status, they pose several challenges, including limited driving range, inadequate charging infrastructure, and the environmental impacts associated with mining and recycling rare earth metals^29–32^. Furthermore, BEVs contribute to non-exhaust emissions, including particulate matter from tire and road wear, a problem exacerbated by their increased weight compared to traditional ICE vehicles^33^.

Additionally, In April 2022, Onorati et al.^34^ studied the role of hydrogen in internal combustion engines (ICEs) and its potential for zero CO_2_ emissions. The study explored how hydrogen-fueled internal combustion engines (H2ICE) can offer a solution for achieving near-zero emissions while retaining the advantages of traditional ICEs. The research reviewed various methods for integrating hydrogen into ICEs, such as direct injection, turbocharging, and lean combustion. With proper after-treatment, they found that H2ICEs could achieve high thermodynamic efficiency, similar to fuel cell powertrains, while producing significantly lower emissions, particularly NO_x_. Moreover, the study noted that hydrogen offers numerous advantages like zero carbon emissions and the ability to use non-purified hydrogen, making it a cost-effective solution for heavy-duty transportation. This research suggests that H2ICEs present a viable alternative to electric powertrains by leveraging existing technologies, with potential benefits for large-scale adoption in both the near and long term.

Furthermore, In June 2023, Duan et al.^35^ studied the combustion characteristics of a turbocharged direct-injection hydrogen engine. The study focused on optimizing performance by investigating how various operational parameters (e.g., equivalence ratio, spark timing, and start of injection) affect the combustion process. The research utilized experimental tests on a 2.0 L turbocharged H2DI, with engine speeds ranging from 1000 to 4000 RPM and loads from 3.7 to 10.6 bar. They found that the direct injection hydrogen engine produced a higher pressure rise rate compared to gasoline and port fuel injection engines. At 1500 rpm, the pressure rise rate reached 3.85 bar/°CA, which was notably higher than other engine types. Additionally, combustion characteristics like burning duration were influenced by factors such as the start of injection and spark timing, with optimized conditions improving brake thermal efficiency to as high as 37.7% at 3000 rpm. While hydrogen combustion reduced emissions of CO and THC, NO_x_ emissions increased under specific conditions. This research suggests that turbocharged direct-injection hydrogen engines offer significant advantages in power density and combustion efficiency, with potential applications in future hydrogen-powered vehicles. The findings contribute to understanding how hydrogen fuel can improve engine performance while addressing emissions concerns.

Additionally, In March 2023, Falfari et al.^36^ studied the potential of hydrogen as a fuel for internal combustion engines (ICEs). The study explored hydrogen’s use in ICEs, focusing on its benefits and challenges. The research utilized a combination of experimental data and computational simulations to analyze hydrogen’s combustion characteristics and behavior. They found that hydrogen, due to its wide flammability limits and high flame speed, provides high efficiency and low emissions in ICEs, although challenges such as abnormal combustion (pre-ignition and backfire) exist. This research suggests that while hydrogen is promising as a sustainable fuel, advancements in injector design and ignition systems are necessary for its effective application in ICEs.

In August 2024, Shahid et al.^37^ extensively reviewed hydrogen production techniques and their application in internal combustion engines (ICEs). The study focused on hydrogen as a sustainable fuel source. It explored various production methods such as water electrolysis (powered by solar and wind energy), biomass gasification, coal gasification, methanol and ammonia decomposition, and steam reforming. These methods aim to reduce reliance on nonrenewable energy sources and improve the sustainability of energy production. The methodology involved reviewing existing literature on hydrogen production, assessing hydrogen’s impact on engine performance, and evaluating emission reductions in both spark-ignition (SI) and compression-ignition (CI) engines. Key findings highlighted that hydrogen enrichment in fuels improved engine performance, increasing thermal efficiency and reducing fuel consumption. In terms of emissions, hydrogen significantly lowered harmful pollutants like HC, carbon monoxide (CO), carbon dioxide (CO_2_), nitrogen oxides (NO_x_), smoke, and soot, especially under optimal operating conditions.

In April 2024, Khalid et al.^38^ studied the factors contributing to backfire in hydrogen port fuel injection internal combustion engines (PFI-H2ICE). The study reviewed strategies for mitigating backfire, a major challenge in these engines . The researchers conducted a literature review that focused on various fuel injection control strategies aimed at optimizing injection timing and hydrogen fuel pressure. They found that inappropriate injection timing, spark timing, and high hydrogen-air concentrations were critical factors causing backfire, which reduced engine performance and potentially damaged components. The review emphasized that delaying the hydrogen injection timing and reducing the hydrogen concentration at the intake valve can effectively mitigate backfire. This research suggests that further optimization of injection control systems will enhance the efficiency and reliability of hydrogen engines in internal combustion systems.

In June 2024, Wittek et al.^39^ studied the full load optimization of a hydrogen-fueled industrial engine. The study focused on optimizing the trade-off between nitrogen oxide (NO_x_) emissions and power output in a converted diesel engine to hydrogen operation. The methodology involved converting a diesel engine to hydrogen combustion and conducting extensive engine tests. The team used techniques such as turbocharging, port fuel injection, and exhaust gas recirculation (EGR) to optimize the engine’s performance while reducing NO_x_ emissions. The authors found that the converted hydrogen engine could exceed the diesel engine’s power output while maintaining low NO_x_ emissions at 1 g/kWh. However, they noted that in the low-end torque range, the hydrogen engine could not fully match the diesel engine’s full load curve. This research suggests that hydrogen-fueled internal combustion engines could offer a feasible alternative to diesel engines, particularly in industrial applications, while contributing to decarbonization efforts by reducing greenhouse gas emissions. This work supports the development of hydrogen engines as a viable carbon-neutral alternative, offering robust power outputs with manageable NO_x_ emissions, thus facilitating cleaner industrial and transportation applications.

In 2023, Bao et al.^40^ investigated the macroscopic spray characteristics of various oxygenated diesel fuels, using high-speed imaging in a constant volume chamber. They found that oxygenated fuels improved spray characteristics, with DMF20 showing the longest penetration and best atomization, which could enhance combustion efficiency and emissions reduction in diesel engines.

In 2025, Cheng Shi et al.^41^ studied the effects of recess geometry modifications on an ammonia-hydrogen Wankel engine, using a 3D CFD model to assess different recess configurations at various compression ratios and speeds. They found that reducing recess dimensions enhanced turbulence, improved flame propagation, and optimized emissions, with CR9.0W at 2000 rpm achieving low NO_x_ and unburned ammonia emissions with minimal efficiency loss. This research highlights the potential of optimizing hydrogen and ammonia-hydrogen combustion strategies for cleaner and more efficient internal combustion engines.

In 2025, Jia et al.^42^ examined the impact of a variable enhanced Miller cycle (VEMC) and exhaust gas recirculation (EGR) on combustion stability in high-compression-ratio gasoline engines, using experimental tests with asynchronous valve control. They found that VEMC with a 90° CA valve spacing angle reduced cycle-to-cycle variations $COV_{IMEP}$ by 36.5% at low loads, while 12% EGR decreased NO_x_ emissions by 68.7% without significant efficiency loss. These results suggest that combining VEMC and EGR can improve both performance and emissions in high-efficiency engines, offering insights for alternative fuel applications like hydrogen combustion.

In 2025, Jia et al.^43^ studied asynchronous variable intake valve phase Miller cycle (AVIVPMC) in a high-CR turbocharged GDI engine. Tests and simulations showed AVIVPMC reduced BSFC by over 5% and improved knock resistance by lowering ECR. However, increased swirl ratio (up to 4.5) weakened tumble flow and combustion efficiency. The work highlights AVIVPMC’s fuel economy benefits but notes trade-offs in combustion quality.

This study makes several significant contributions to the field of hydrogen-fueled internal combustion engine (H2ICE) research, specifically through the use of 1D modeling and simulation. Key contributions include:

- **Performance and emissions insights:** Provides a detailed evaluation of hydrogen combustion characteristics, showing its potential for significantly reducing CO emissions while addressing challenges such as elevated NO_x_ emissions due to high combustion temperatures.
- **Water injection strategy:** Demonstrates the effectiveness of water injection in mitigating NO_x_ formation, improving volumetric efficiency, and balancing emissions with performance metrics across various hydrogen-to-water ratios.
- **Optimization parameters:** Identifies optimal operational conditions, including air-fuel ratio ($\lambda$), spark timing, and water injection levels, that maximize efficiency and minimize environmental impact, paving the way for practical H2ICE applications.
- **Comparative analysis:** Offers a comprehensive comparison of gasoline, hydrogen, and hydrogen-with-water injection engine models, showcasing trade-offs and opportunities for hydrogen as a sustainable alternative fuel.
- **Simulation validation:** Utilizes a validated Ricardo WAVE simulation model, ensuring the reliability of findings and their applicability to real-world scenarios.

This study presents a detailed 1D simulation of a hydrogen-fueled internal combustion engine, employing advanced strategies such as water injection to address NO_x_ emissions while maintaining performance. Through systematic optimization of the air-fuel ratio, spark timing, and hydrogen-to-water ratios, the work provides critical insights into achieving efficient and low-emission hydrogen combustion. The validated model, benchmarked against manufacturer data, offers a robust framework for evaluating hydrogen’s viability as a carbon-neutral fuel. By comparing gasoline, hydrogen, and hydrogen-with-water injection configurations, the analysis shows key trade-offs in power, efficiency, and emissions, contributing actionable solutions for sustainable engine design and decarbonization efforts.

The paper is organized as follows: the methodology outlines the 1D modeling approach and simulation setup, followed by the results and discussion, which evaluate key performance metrics under varying operational conditions. Finally, the conclusion summarizes the findings and proposes future research directions for hydrogen-based propulsion systems.

## Methodology

### Overview

A 1D engine model is a computational tool used to simulate unsteady airflow within an engine, providing insights into pressure pulsations, flow losses, and their impact on the torque curve. The model represents the engine’s flow system as a network of ducts, volumes, valves, and orifices. Pipes are modeled as one-dimensional elements defined by their length and diameter, while cylinders are treated as zero-dimensional volumes that change over time. The model solves equations for momentum, energy, and mass conservation, while empirical data account for flow losses and combustion heat release.

Combustion in the cylinder is modeled by simulating the heat release from the air-fuel mixture, which directly affects cylinder pressure and torque output. Mechanical friction (FMEP) is modeled using empirical data, while pumping losses (PMEP) are calculated based on gas pressures. Although some aspects, like flow losses and combustion profiles, rely on empirical inputs, the model’s prediction of unsteady airflow is more physics-based and accurate.

For hydrogen engine conversion, 1D modeling helps evaluate airflow dynamics, heat release, and torque behavior to optimize performance. Ricardo WAVE 2019.1^44^, a leading 1D simulation software, supports these analyses. It models pressure waves, mass flow, and energy losses in ducts and manifolds while providing elements like engine cylinders, compressors, and turbochargers. Widely used in industries such as automotive, motorsport, and power generation, WAVE enables performance simulations for various intake, combustion, and exhaust system configurations^45^.

### Engine selection

A single-cylinder motorcycle engine was chosen because it simplifies the task of adding custom subsystems such as hydrogen fueling, water injection, and ignition control. Having single cylinders reduces mechanical and electrical complexity, making it easier to install sensors, harnesses, and other components. Ultimately, the single-cylinder design balances simplicity, adaptability, and reliability, making it ideal for a prototype conversion.

**Fig. 1:**
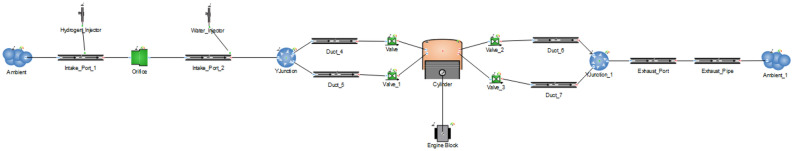
1D engine model arrangements.

The selected engine for this project is the BMW F650 GS engine shown in Fig. 1, which is a reliable single-cylinder engine known for its balance of performance. Its specifications, detailed in Table 1

**Table 1:** BMW F650 GS engine specifications.

Specification	Value
Engine type	Single-cylinder, 4-stroke
Displacement	652 cc
Cooling system	Liquid-cooled
Bore × Stroke	100 mm × 83 mm
Compression ratio	11.5:1
Maximum power	50 HP (37 kW) @ 6500 rpm
Maximum torque	60 Nm @ 5000 rpm
Starter system	Electric starter
Transmission	5-speed manual
Lubrication	Dry sump, with oil pump
Valve configuration	DOHC, 4 valves

### Engine sub-models

Engine submodels are essential for simulating and analyzing how internal combustion engines work. These models represent key processes like piston motion, combustion, heat transfer, Emissions, Knock, and valve operation. Tools like Ricardo WAVE use these submodels to predict engine performance and behavior under different conditions accurately. Each submodel focuses on a specific part of the engine’s operation, helping engineers improve engine design and performance with reliable and detailed simulations.

### Crank-slider piston motion

The crank/slider sub-model is used to characterize the arrangement of mechanical parts designed to convert translational motion into rotational motion or vice-versa. The piston motion is thus defined by including geometric inputs for the cylindrical combustion chamber, crankshaft, and connecting rod. Ultimately, the piston position is required to calculate the combustion chamber’s volume.

The total displacement of each cylinder is calculated as:

1 $$\begin{aligned} \text {Disp} = \frac{\pi }{4} \cdot \text {Bore}^2 \cdot \text {Stroke} \end{aligned}$$

The total engine displacement is then calculated by summing the displacements of all cylinders and displayed in the locked input field labeled “Displacement”.

Using this displacement, the volume at TDC (clearance volume) is calculated using the compression ratio in the following equation:

2 $$\begin{aligned} Vol_{TDC} = \frac{Disp}{(CR - 1)} \end{aligned}$$

Then, the instantaneous volume of the cylinder can be calculated on a per-timestep basis using the following equation:

3 $$\begin{aligned} Vol = Vol_{\text {TDC}} + \frac{\pi }{4} \cdot \text {Bore}^2 \cdot s \end{aligned}$$

where: s = Piston position in reference to its TDC position, with positive being away from its TDC position

The piston position is calculated using a standard crank/slider calculation as shown in Fig. 1. As default 0 deg refers to the piston position at TDC.

4 $$\begin{aligned} s = \sqrt{((a + 1)^2 - \text {pinoff}^2)} - a \cos \theta - \sqrt{1^2 - (a \sin \theta + \text {pinoff})^2} \end{aligned}$$

where: *a* = Crank radius (half the stroke)

l = Connecting rod length

pinoff = Wrist pin offset

*q* = Crank angle from TDC

### Combustion model

The engine model simulates in-cylinder processes by solving mass and energy equations over time. The mass equation tracks air, fuel (both liquid and vapor), and combustion products entering and exiting the cylinder. Liquid fuel is considered for its mass but occupies minimal volume due to its high density. The energy equation, based on the first law of thermodynamics, accounts for internal energy changes through enthalpy flux, heat transfer, and piston work.

For combustion, the model uses the SI Wiebe function to simulate the fuel burn rate in spark-ignition engines. This function, widely used in single-fuel engine simulations, allows independent control of burn duration and shape parameters, accurately reflecting experimental combustion behavior. It provides a mathematical representation of the fuel burn process relative to the crank angle, making it a standard tool for predicting SI engine performance.

5 $$\begin{aligned} W = 1.0 - \textrm{exp}\left( -AWI\left( \frac{\theta }{BDUR}\right) ^{[\text {WEXP}+1]}\right) \end{aligned}$$

*AWI* = Internally calculated parameter to allow BDUR to cover the range of 10–90% , *q* = Degrees past the start of combustion , *BDUR* = User-entered combustion duration (10–90%) , and *WEXP* = User-entered exponent in Wiebe function

The burn profile in the input panel allows analysis of combustion parameter variations. Adjusting the 50% burn point shifts the curve forward or backward, while modifying the 10–90% duration changes the overall burn time. The Wiebe exponent influences whether combustion occurs earlier or later in the cycle.

For dual-fuel models like hydrogen with water injection, the Multi-component Wiebe Combustion Model is used. This advanced model combines up to eight Wiebe curves, simulating complex burn profiles, including single, double, or triple Wiebe configurations. It supports both premixed (homogeneous) and non-premixed (spray-guided) combustion.

When a multi-fuel file is loaded, the model automatically applies multi-fuel combustion settings, assigning each fuel as either Premixed or Non-Premixed based on its mixing behavior. The model assumes two combustion zones-premixed and non-premixed-each occupying part of the cylinder air.

In hydrogen with water injection, both components use premixed combustion for accurate simulation. This flexibility makes the Multi-component Wiebe model ideal for complex engine systems with advanced fuel strategies.

Assume there are $N_p$ fuels (with each of mass $F_{p,i}$ ) premixed with air (with mass $A_p$). The equivalence ratio of combustion for each fuel is assumed constant and given by

6 $$\begin{aligned} \phi _p = \sum _{i=1}^{N_p} \text {AFS}_{p,i} \frac{F_{p,i}}{A_p} \end{aligned}$$

where $AFS_{p,i}$ is the stoichiometric air-fuel ratio of fuel. The above formula applies to the premixed fuels in the whole cylinder.

In the Premixed zone, the premixed fuel mass to be burned:

7 $$\begin{aligned} F_{p_i}\big |_{\text {Zone1}} = f_{\text {air}\_\text {z1}} F_{p_i} \end{aligned}$$

Note that air mass in this zone is always

8 $$\begin{aligned} A|_{Zone1} = f_{air_{z1}} A \end{aligned}$$

where *A* is the air mass in the whole cylinder. $f_{air_{z1}}$ is the air fraction for the Premixed Zone.

For a given time step $\Delta t$, $\Delta w_{\text {premix}}$ fuel fraction burned is given by the Premixed Combustion profile. The fuel mass burned can be calculated from

9 $$\begin{aligned} \left. \Delta F_{p,i} \right| _{\text {Zone1}} = \Delta w_{\text {premix}} \times \left. F_{p,i} \right| _{\text {Zone1}} \end{aligned}$$

The corresponding air mass burned for the step can be calculated from

10 $$\begin{aligned} \Delta A \big |_{\text {zone1}} = \frac{\sum \text {AFS}_{p_i} \times \Delta F_{p_i} \big |_{\text {zone1}}}{\phi _p} \end{aligned}$$

For complete combustion, the following by summing up all steps of combustion,

11 $$\begin{aligned} \sum \Delta A \big |_{Zone1} = A \big |_{Zone1} \end{aligned}$$

### CO emissions

The CO emissions sub-model predicts CO production during combustion and exhaust in an engine cylinder element.

For lean combustion, the full equilibrium calculation under-predicts engine-out CO concentrations by several orders of magnitude. In order to efficiently predict emission levels, the CO concentrations are recalculated in parallel with the in-cylinder thermodynamic calculation. The following procedure is used:

At each step during combustion, the mole fractions of WAVE’s eleven species are calculated for the unburned and burned zones based on thermodynamic equilibrium and then averaged. The single-zone model is used when combustion ends. The crank angular position at which the CO mole fraction reaches its maximum value is then determined.

As known, the recombination reactions of H and OH species are the third-body reactions:

12 $${\text{H}} + {\text{HO}} + {\text{M}} \to {\text{H}}_{2} {\text{O}} + {\text{M }}$$

13 $${\text{H}} + {\text{H}} + {\text{M}} \to {\text{H}}_{2} + {\text{M}}$$

The third-body reaction rates are very slow, hence a sudden freezing of species H and OH when CO reaches a maximum is suggested by Newhall^46^.

he rest of the nine species are thus calculated from thermodynamic equilibrium and atomic number conservation of elements H, C, O and N plus the following constraint on CO and CO_2_. The chemical reaction originally used in the WAVE’s gas property calculation gives good results for fuel rich mixture combustion, but poor predictions for fuel lean mixture combustion:

14 $$\begin{aligned} & \text {CO} + \frac{1}{2} \text {O}_2 \rightarrow \text {CO}_2 \end{aligned}$$

15 $$\begin{aligned} & \frac{[\text {CO}]}{[\text {CO}_2]} = \frac{1}{\sqrt{K_pw P[\text {O}_2]}} \end{aligned}$$

where: $K_pw$ = Equilibrium constant of the reaction as used in WAVE’s gas property calculation

P = Pressure

However, the chemical reaction suggested by Newhall^46^ gives good results for lean combustion, but poor predictions for rich combustion:

16 $${\text{CO}} + {\text{OH}} \to {\text{CO}}_{2} + {\text{H}}$$

17 $$\begin{aligned} & \frac{[\text {CO}]}{[\text {CO}_2]} = \frac{1}{K_{pN}} \frac{[\text {H}]}{[\text {OH}]} \end{aligned}$$

where: $K_{pN}$ = Equilibrium constant of the reaction as suggested by Newhall

Therefore, these two reactions are combined to cover the whole fuel concentration range. In general, the CO and CO_2_ concentrations of the combustion products in the rich mixture side are dictated by the O2 concentration, while in the lean mixture side they are dictated by the H and OH concentrations. Thus, we can determine the concentration ratio of CO to CO_2_ for the whole fuel concentration range, as below,

18 $$\begin{aligned} \frac{[\text {CO}]}{[\text {CO}_2]} = \max \left\{ \frac{1}{\sqrt{K_{pW} P[\text {O}_2]}},\frac{1}{K_{pN}} \frac{[\text {H}]}{[\text {OH}]}\right\} \end{aligned}$$

This procedure has been used for comparison with measurements. Tests have shown that the computed and measured engine out CO concentrations are within one order of magnitude.

### NO_x_ emissions

The NO_x_ emissions sub-model predicts NO_x_ production during combustion and exhaust in an engine cylinder element. it uses the chemistry of all fuels in the cylinder to predict NO_x_ production.

For accurate treatment of NO_x_ kinetics, which are strongly temperature dependent, the non-homogeneity of the temperature field within the combustion chamber must be taken into account. Thus, the NO_x_ emissions sub-model requires specification of the 2- zone combustion thermodynamics. At any instant during the combustion process, there is mass flux into the burned zone associated with the instantaneous fuel burning rate and the stoichiometry of the incremental burned mass (packet). The NO_x_ model assigns an initial NO_x_ concentration to each packet representing the prompt and residual NO_x_. During combustion, the packets that burn early in the cycle are compressed for a longer period so that they attain a higher temperature, thus contributing more NO_x_ than those which burn later.

The NO_x_ model accounts for the “prompt” or “flame-formed” NO, which is due to the over-equilibrium radical concentration (oxygen atom and hydroxyl radical) in the flame region. The value of the prompt NO is obtained from the correlation of the data reported by Fenimore^47^ which gives the ratio of prompt NO to equilibrium NO as a function of equivalence ratio. All the NO_x_ is assumed to be in the form of NO during the prompt formation phase as well as the thermal phase described below by the extended Zeldovich mechanisms of NO_x_ formation:

19 $${\text{N}} + {\text{NO}}_{2} \to {\text{N}}_{2} + {\text{O}}$$

20 $${\text{N}} + {\text{O}}_{2} \to {\text{NO}} + {\text{O}}$$

21 $${\text{N}} + {\text{OH}} \to {\text{NO}} + {\text{H}}$$

The overall burned zone is treated as an open, stratified system in which further NO_x_ formation takes place depending on the temperature, pressure, and equivalence ratio of the burned packet

thermodynamics equilibrium values are used for the species O_2_, O, H, and OH. The steady-state assumption is used for highly reactive N atoms. The concentration of NO versus time is solved using an open system in which the above elementary reactions are used with those rate constants reported by Heywood^48^ For the first reaction equation, the rate constant, $R_1$, is given by:

22 $$\begin{aligned} R_1 = A \cdot ARC1 \cdot e^{({T_a \cdot AERC1 }/{T})} \end{aligned}$$

For the second and third reaction equations, the rate constant, $R_{2/3}$ , is given by:

23 $$\begin{aligned} R_{2/3} = A \cdot e^{({T_a}/{T})} \end{aligned}$$

where:

A = Pre-exponential constant , ARC1 = User-entered pre-exponent multiplier , $T_a$ = Activation temperature for the reaction , AERC1 = User entered exponent multiplier , and T = Burned-zone temperature

The calculation is terminated when the temperature in the burned zone reaches a low enough level so that the kinetics become inactive and total NO no longer changes.

### Knock

The simple knock sub-model is based on the Douaud and Eyzat (1978)^49^ induction time correlation.

Here is how the software predicts knocking:

Firstly, The induction time (ignition delay) in seconds is calculated at every timestep using the following equation:

24 $$\begin{aligned} \tau = 0.01869 / A_{p} \left( \frac{ON}{100} \right) ^{3.4107} \cdot P^{-1.7} \textrm{exp}\left( \frac{3800 / A_{T}}{T} \right) \end{aligned}$$

where:

$A_P$ = User-entered pre-exponential multiplier , ON = User-entered fuel octane number , P = Cylinder pressure [kgf/cm ] , $A_T$ = User-entered activation temperature multiplier , and T = Unburned gas temperature [K]

In general, this induction time continually decreases as combustion progresses and the unburned zone temperature rises. The endgas auto-ignites (knocks) if the induction time is less than the flame arrival time.

The model assumes that auto-ignition occurs when:

25 $$\begin{aligned} \int _{t_0}^{t_i} \frac{dt}{t} = 1 \end{aligned}$$

where:

$t_0$ = Start of end-gas compression , $t_i$ = Time of auto-ignition , and t = Induction time, defined above

when knock occurs, a spontaneous mass burning rate due to knock is determined and fed back to the cylinder, leading to a rapid rise in cylinder pressure and temperature. The in-cylinder heat transfer coefficient is also increased during knock.

Combustion is then governed by the post-knock burn time scale as shown below:

26 $$\begin{aligned} \tau _{postknock} = f_{\tau } \left[ \frac{0.8573}{B_{o}(1 + A/F)} \textrm{exp}\left( \frac{T_{a}}{T_{\tau }} \right) \right] \end{aligned}$$

where:

$f_t$ = Post-knock burn scale multiplier , $B_0$ = Frequency factor, hard-coded as 2233e3 1/s , A/F = Air/fuel ratio of the unburned end gas , $T_a$ = Activation temperature, hard-coded as 15150 K , and $T_f$ = Adiabatic flame temperature

The fuel burn rate in the post-knock period is assumed to be constant and is calculated as:

27 $$\begin{aligned} \dot{m}_{dot,fuel} = \frac{m_{f,vapor} + m_{f,liquid}}{\tau _{postknock}} \end{aligned}$$

Air is burned proportionally at a rate given by:

28 $$\begin{aligned} \dot{m}_{dot, air} = \dot{m}_{dot, fuel} * \left( \frac{A}{F} \right) \end{aligned}$$

where:

$m_{f,vapor}$ = Unburned fuel vapor mass at the time of knock , and $m_{f,liquid}$ = Unburned fuel liquid mass at the time of knock

### Conduction & heat transfer

Conduction sub-models are used to calculate in-cylinder surface temperatures. Accurate surface temperatures improve the boundary conditions for the in-cylinder heat transfer sub-models and can be used to assist in engine component design.

the convective heat transfer coefficient was predicted using the Woschni correlation^50^. The Woschni heat transfer sub-model views the charge as having a uniform heat flow coefficient and velocity on all cylinder surfaces and calculates the amount of heat transferred to and from the charge based on these assumptions.

The Woschni heat transfer coefficient is calculated using the following equation:

29 $$\begin{aligned} h_z = 0.0128D^{-0.20}P^{0.80}T^{-0.53}v_c^{0.8}C_{enht} \end{aligned}$$

where:

D = Cylinder bore , P = Cylinder pressure , T = Cylinder temperature , $V_c$ = Characteristic velocity , and $C_{enht}$ = User-entered multiplier

The characteristic velocity is the sum of the mean piston speed and an additional combustion-related velocity that depends on the difference between the cylinder pressure and the pressure that would exist under motoring conditions. It is given by Woschni’s original correlation^50^as:

30 $$\begin{aligned} v_c = c_1v_m + c_2\frac{V_DT_r}{P_rV_r}(P - P_{mot}) \end{aligned}$$

or by Woschni’s modified correlation^51^, which includes a load compensation term, as:

31 $$\begin{aligned} v_c = \max \left[ \left( c_1v_m + c_2\frac{V_DT_r}{P_rV_r}(P - P_{net}) \right) , \left( c_1v_m \left( 1 + 2 \left( \frac{V_c}{V} \right) ^2 IMEP^{-0.2} \right) \right) \right] \end{aligned}$$

where:

$v_m$ = Mean piston speed , $V_D$ = Cylinder displacement , $T_r$ = Reference temperature , $P_r$ = Reference pressure , $V_r$ = Reference volume , $P_{mot}$ = Motored cylinder pressure , $V_c$ = Clearance volume , *V* = Instantaneous cylinder volume , and IMEP = Cylinder indicated mean effective pressure

The coefficient, c is a dimensionless quantity calculated as:

During scavenging:

32 $$\begin{aligned} c_1 = 6.18 + 0.417\frac{v_s}{v_m} \end{aligned}$$

When valves are closed:

33 $$\begin{aligned} c_1 = 2.28 + 0.308\frac{v_s}{v_m} \end{aligned}$$

with the swirl velocity, $v_s$, calculated from a user-entered or predicted swirl ratio:

34 $$\begin{aligned} v_s = \pi * R_{swirl} * D * \frac{RPM}{60} \end{aligned}$$

The coefficient, c is a constant given as:

During combustion:

35 $$\begin{aligned} c_2 = 3.24 \times 10^{-3} \left[ \frac{m}{s * K} \right] \end{aligned}$$

Before combustion and during scavenging:

36 $$\begin{aligned} c_2 = 0 \end{aligned}$$

Once the discretization process was completed, every part was exported to the Ricardo-Wave software. In this module, the 1D converted parts were arranged accordingly, starting from the ambient air until the exhaust tailpipe. Every part was connected using ducts as shown in Fig. 1 Then, using the engine technical specifications as shown in Table 1, and the temperature for engine parts in Ricardo-Wave software as shown in Table 2, detailed configurations were set for every parameter, and engine components were involved. Valves diameters, wall temperatures, flow arrays, and valve opening and closing durations were taken into serious consideration.

**Table 2:** Engine parts and their temperatures (K).

Engine part	Temperature (K)
Piston	525
Liner	500
Head	550
Intake valve	400
Exhaust valve	450

The next thing was setting different air-fuel ratios, spark timing, and various amounts of water injection for different engine speeds and the separation of several cases.

The simulation runs according to the cases set so that, the output performance can be compared to the engine’s service manual and with each model results which run with different configurations of air-fuel ratios, spark timing, and various amounts of water injection for every different engine speed. The output from the successfully performed simulation was shown in R-Post. The results were collected and tabulated to be used for the validation, comparison, and optimization process by using MATLAB R2023b^52^.

## Results and discussion

To analyze the effect of hydrogen different air-fuel ratios, spark timing, and amount of water injection on the performance of the hydrogen port fuel injection engine, five different studies have been used.

- **Phase 1**: Comparison and validation of gasoline simulation engine model with real gasoline engine based on the percentage difference in engine performance data in the manufacturer’s manual.
- **Phase 2**: Convert gasoline fuel to hydrogen in the engine model and compare results with gasoline and hydrogen.
- **Phase 3**: The hydrogen-fueled model was optimized by adjusting various parameters derived from the Design of Experiments (DOE) module. The simulation was then rerun using these optimized parameters.
- **Phase 4**: In the hydrogen-fueled model, varying amounts of water injection were introduced to analyze their impact on engine performance and emissions. The simulation results were evaluated to determine changes in parameters such as power, torque, thermal efficiency, NO_x_, and other pollutants’ emission levels. These results provided valuable insights into water injection’s potential benefits and trade-offs in hydrogen-fueled engines.
- **Phase 5**: comparing the gasoline model, the optimized hydrogen model, and the hydrogen model with the optimum amount of water injection across various engine performance metrics.

### Gasoline model validation

The primary goal of this phase is to validate the accuracy of the gasoline engine simulation model by comparing its results with the performance data provided in the manufacturer’s service manual. This ensures the model can reliably predict engine performance before proceeding to hydrogen conversion and optimization.

The gasoline simulation model was validated using the manufacturer’s service manual data for brake power (50 hp @ 6500 rpm) and brake torque (60 Nm @ 5000 rpm). The manual provides only these two data points, shown in Table 3. The simulation was run from 2000 to 7500 rpm over 30 cycles, and the results were exported to Excel 2016^53^, plotted using Matlab R2023b^52^, and used to predict additional performance metrics (e.g., BMEP, BSFC, exhaust temperature, volumetric efficiency, and emissions). These predicted values, along with the brake power and torque validation, serve as the baseline for comparison in Phases 2, 3, and 4, where the engine is converted to hydrogen and optimized with water injection.

**Table 3:** Manufacturer’s service manual data for brake power and brake torque.

Power output kW (hp)	37 (50) @ 6500 rpm
Max. torque Nm	60 @ 5000 rpm

**Fig. 2:**
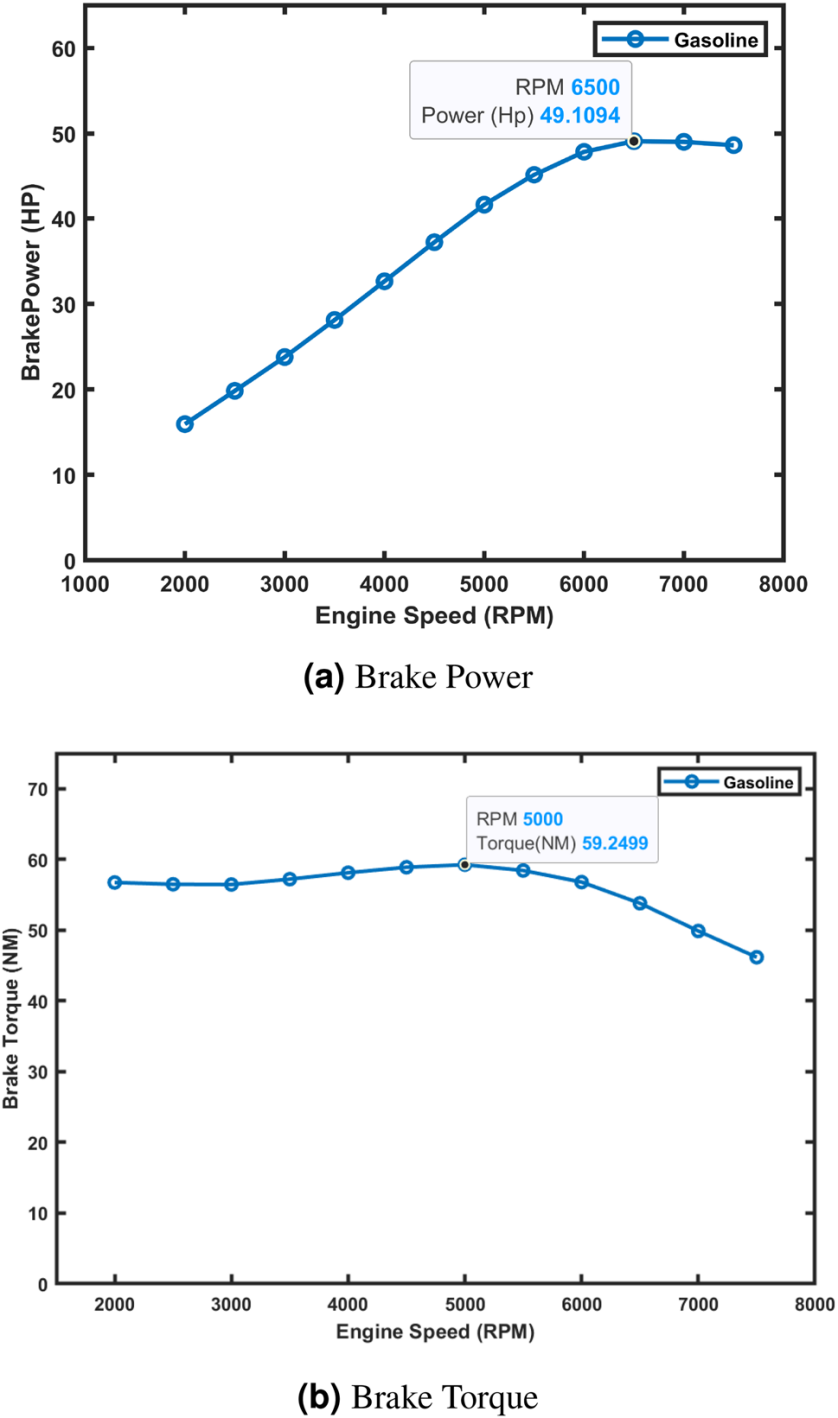
Brake power and torque curves.

The simulation model was evaluated across the entire operating range (2000–7500 rpm) and compared to manufacturer data at key operating points. At 6500 rpm, the simulated brake power was 49.11 hp, deviating by only 1.78% from the manufacturer’s value of 50 hp. Similarly, at 5000 rpm, the simulated brake torque was 59.25 Nm, within 1.08% of the manufacturer’s specified 60 Nm. Figure 2a and b display the full brake power and torque curves, respectively, with the manufacturer’s data points highlighted for comparison.

These results demonstrate the simulation model’s accuracy in replicating engine performance and its reliability for predicting behavior across the rpm range.

**Fig. 3:**
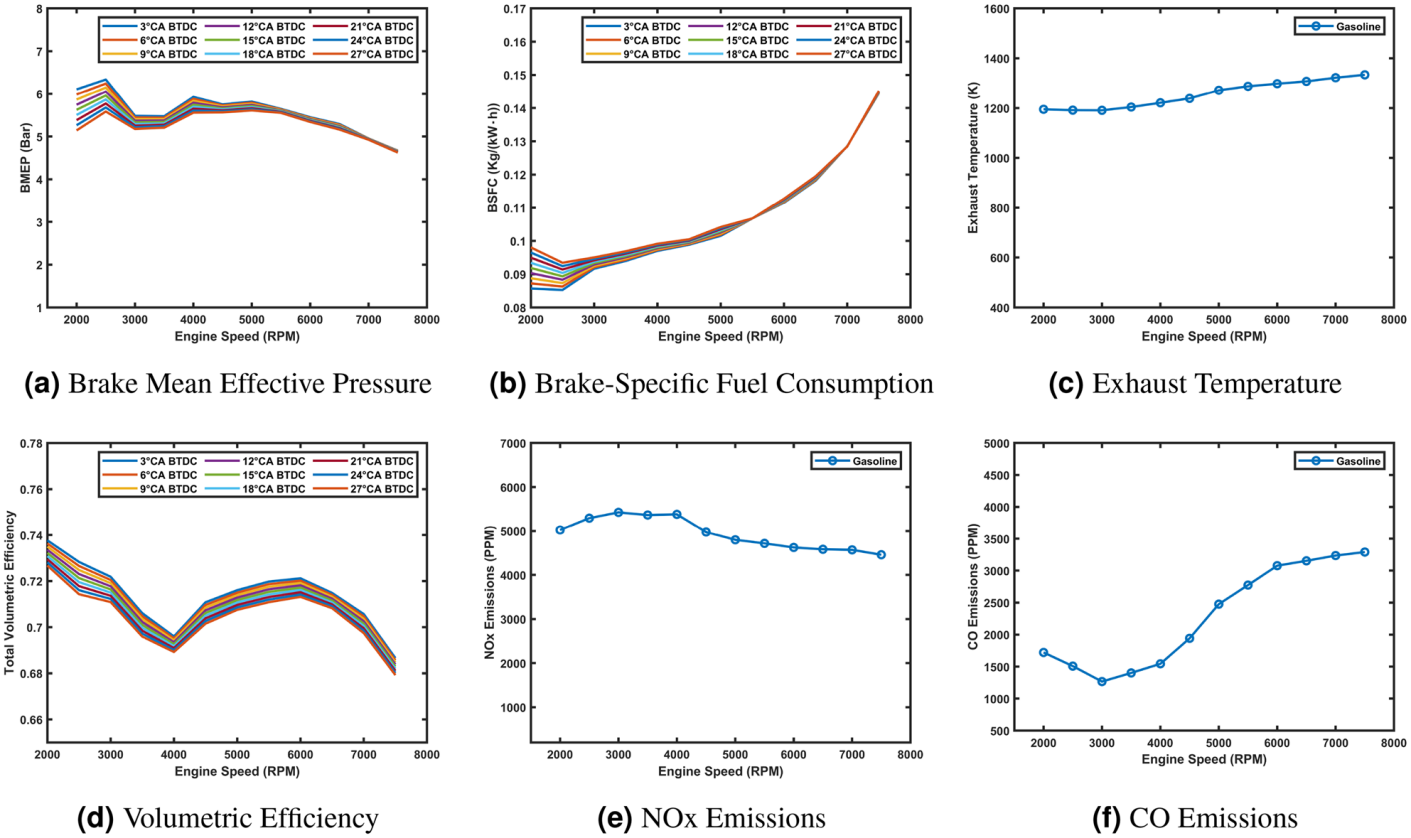
Performance and emissions characteristics of the engine across the operating range.

The simulated engine performance across the operating range (2000–7500 rpm) is summarized as follows: **BMEP** peaks at 11.4 bar around 5000 rpm (Fig. 3a), indicating the engine’s most efficient operating point for mechanical work. **BSFC** reaches a minimum of 0.22 kg/kWh at 3000 rpm (Fig. 3b), highlighting optimal fuel efficiency. **Exhaust temperature** increases with rpm, peaking at 1334 K at 7500 rpm (Fig. 3c), providing insights into thermal behavior and emissions characteristics. **Volumetric efficiency** peaks at 95.8% around 5000 rpm (Fig. 3d), indicating efficient air intake. **NO**_**x**_ emissions peak at 5380 ppm at 4000 rpm (Fig. 3e), while **CO** emissions reach a maximum of 3390 ppm at 7500 rpm (Fig. 3f). These results offer a detailed understanding of engine performance and emissions behavior, serving as a baseline for comparing the gasoline model with hydrogen and hydrogen-with-water configurations.

The validation of the gasoline simulation model against manufacturer data confirms its accuracy, with close agreement in brake power and torque. Predicted metrics for BMEP, BSFC, exhaust temperature, volumetric efficiency, and emissions provide a comprehensive baseline for understanding the engine’s performance and emissions characteristics. These results form a solid foundation for the next phases of the study, focusing on the engine’s conversion to hydrogen fuel and optimization with water injection, enabling evaluation of their impacts on performance and emissions.

### Hydrogen fueled model

The gasoline engine model was converted to operate on hydrogen fuel, and the results were compared with the baseline gasoline model. Both models were simulated using their respective stoichiometric air-fuel ratios (14.7 for gasoline and 34.3 for hydrogen). The performance and emissions characteristics of the two models were analyzed across the operating range of 2000–8000 rpm, and the results are presented in the following discussion, supported by the plotted graphs.

**Fig. 4:**
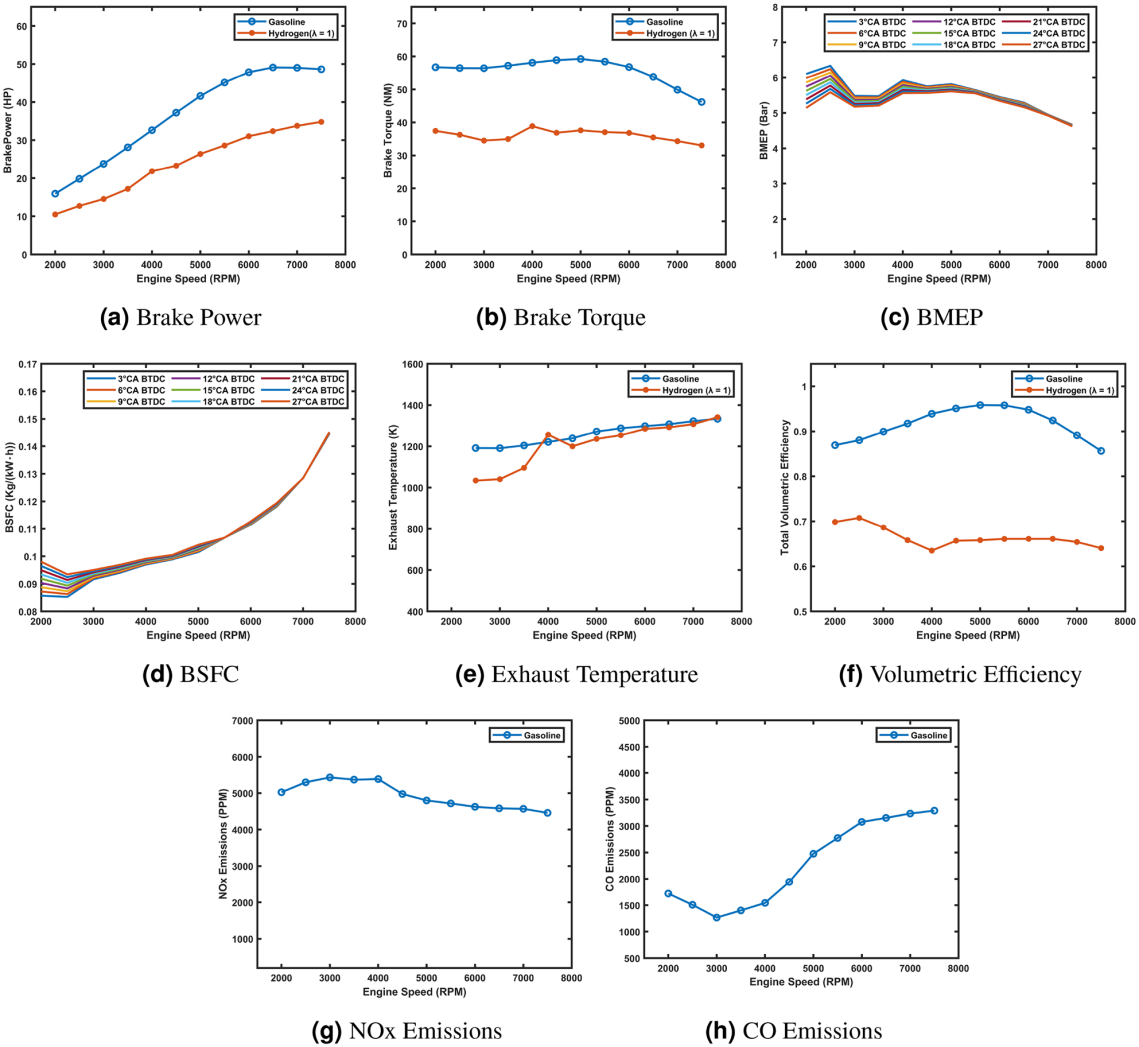
Comparison of hydrogen and gasoline engine performance and emissions characteristics.

The hydrogen-fueled engine showed reduced performance compared to the gasoline model, with approximately 28% lower brake power and 34% lower brake torque (Fig. 4a and b) due to hydrogen’s lower energy density and the engine’s gasoline-optimized design. Similarly, BMEP was 34% lower (Fig. 4c), reflecting reduced peak cylinder pressures. However, hydrogen demonstrated better fuel efficiency, with a 63% lower BSFC at 2500 rpm (Fig. 4d), and lower exhaust temperatures, approximately 100 K lower at 6500 rpm (Fig. 4e), due to higher water vapor production. Volumetric efficiency was consistently lower (Fig. 4f), limited by hydrogen’s lower density. Emission-wise, NO_x_ levels were about 20% higher at 4500 rpm (Fig. 4g) due to higher combustion temperatures, while CO emissions were negligible (Fig. 4h) due to hydrogen’s carbon-free combustion. These results highlight key trade-offs and opportunities for optimizing hydrogen-fueled engines.

The conversion of the gasoline engine model to hydrogen fuel revealed key trade-offs in performance and emissions. While hydrogen offers benefits such as reduced BSFC and negligible CO emissions, it also results in lower brake power, torque, and BMEP, along with higher NO_x_ emissions. These findings highlight the need for further optimization to address these challenges. This phase provides valuable insights into hydrogen’s potential as an alternative fuel for internal combustion engines, forming a foundation for future research and development.

### Influence of air-fuel ratio (AFR) on engine performance and emissions

shows the effects of varying air-fuel ratios (Lambda, $\lambda$) on the performance and emissions of a hydrogen-fueled port injection engine. Key performance metrics such as Brake Mean Effective Pressure (BMEP), brake torque, brake power, Brake Specific Fuel Consumption (BSFC), exhaust gas temperature, heat transfer rate, in-cylinder pressure, NO_x_ emissions, and volumetric efficiency are analyzed. The engine simulations were conducted through 30 engine cycles across a speed range of 2000 to 7500 RPM and Lambda values range from 1.4 to 3. The goal is to understand how the air-fuel ratio influences engine performance and to identify the optimal Lambda value that balances power, efficiency, and emissions.

**Fig. 5:**
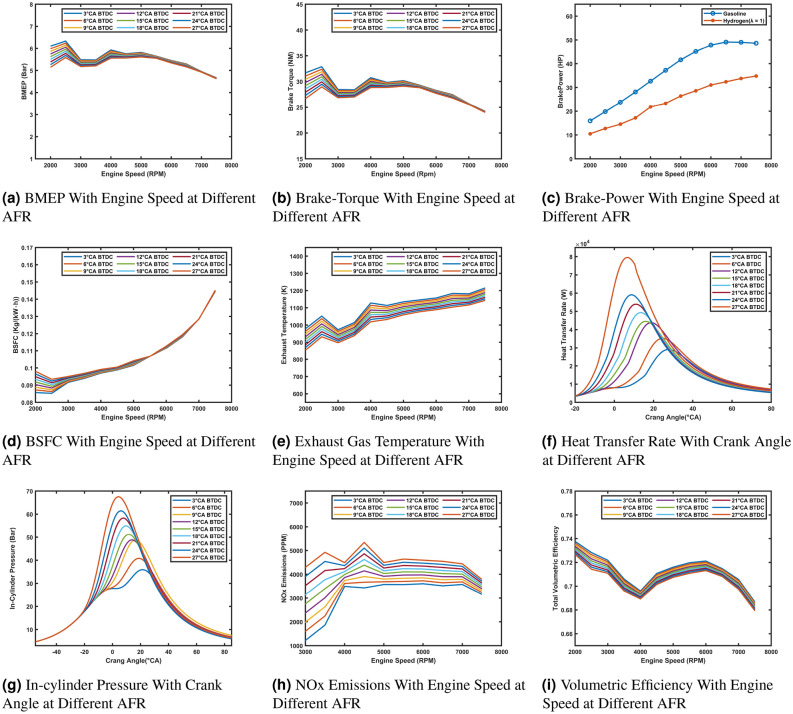
Comparison of engine performance and emissions at different AFR values.

The effect of lambda on engine performance and emissions highlights the critical role of air-fuel ratio in combustion. BMEP (Fig. 5a) peaks at $\lambda$ = 1.4, reflecting optimal energy release from near-stoichiometric combustion. Beyond $\lambda$ = 2.0, leaner mixtures reduce available hydrogen, causing significant drops in BMEP, brake torque (Fig. 5b), and brake power (Fig. 5c). BSFC (Fig. 5d) is lowest at $\lambda$ = 1.4 and increases as $\lambda$ rises due to incomplete combustion and pumping losses.

Exhaust temperatures (Fig. 5e) are highest at $\lambda$ = 1.4 and decrease with leaner mixtures, particularly beyond $\lambda$ = 2.4. Heat transfer rates (Fig. 5f) follow a similar trend, peaking at $\lambda$ = 1.4 and declining with increasing lambda. In-cylinder pressures (Fig. 5g) peak at $\lambda$ = 1.4 ( 55 bar) due to rapid energy release, while higher lambda values show delayed and less efficient combustion.

NO_x_ emissions (Fig. 5h) are highest at $\lambda$ = 1.4 and decrease with leaner mixtures due to lower flame temperatures, while ultra-lean mixtures ($\lambda$ = 3) produce negligible emissions. Volumetric efficiency (Fig. 5i) peaks at $\lambda$ = 1.4 and decreases as lambda increases, reflecting reduced density and higher residual gases. These findings highlight the importance of lambda control in optimizing engine performance and balancing power, efficiency, and emissions.

### Influence of spark timing on engine performance and emissions

This subsection shows the effects of varying Spark-Timing on the performance- mance and emissions of a hydrogen-fueled port injection engine. Key performance metrics such as Brake Mean Effective Pressure (BMEP), brake torque, brake power, Brake Specific Fuel Consumption (BSFC), exhaust gas temperature, heat transfer rate, in-cylinder pressure, NO_x_ emissions, and volumetric efficiency are analyzed. The engine simulations were conducted through 30 engine cycles across a speed range of 2000 to 7500 RPM with lambda value equal to 1.4 and spark timing values ranging from 3°CA BTDC to 27° CA BTDC. The goal is to understand how the spark timing influences engine performance and to identify the optimal timing value that balances power, efficiency, and emissions.

**Fig. 6:**
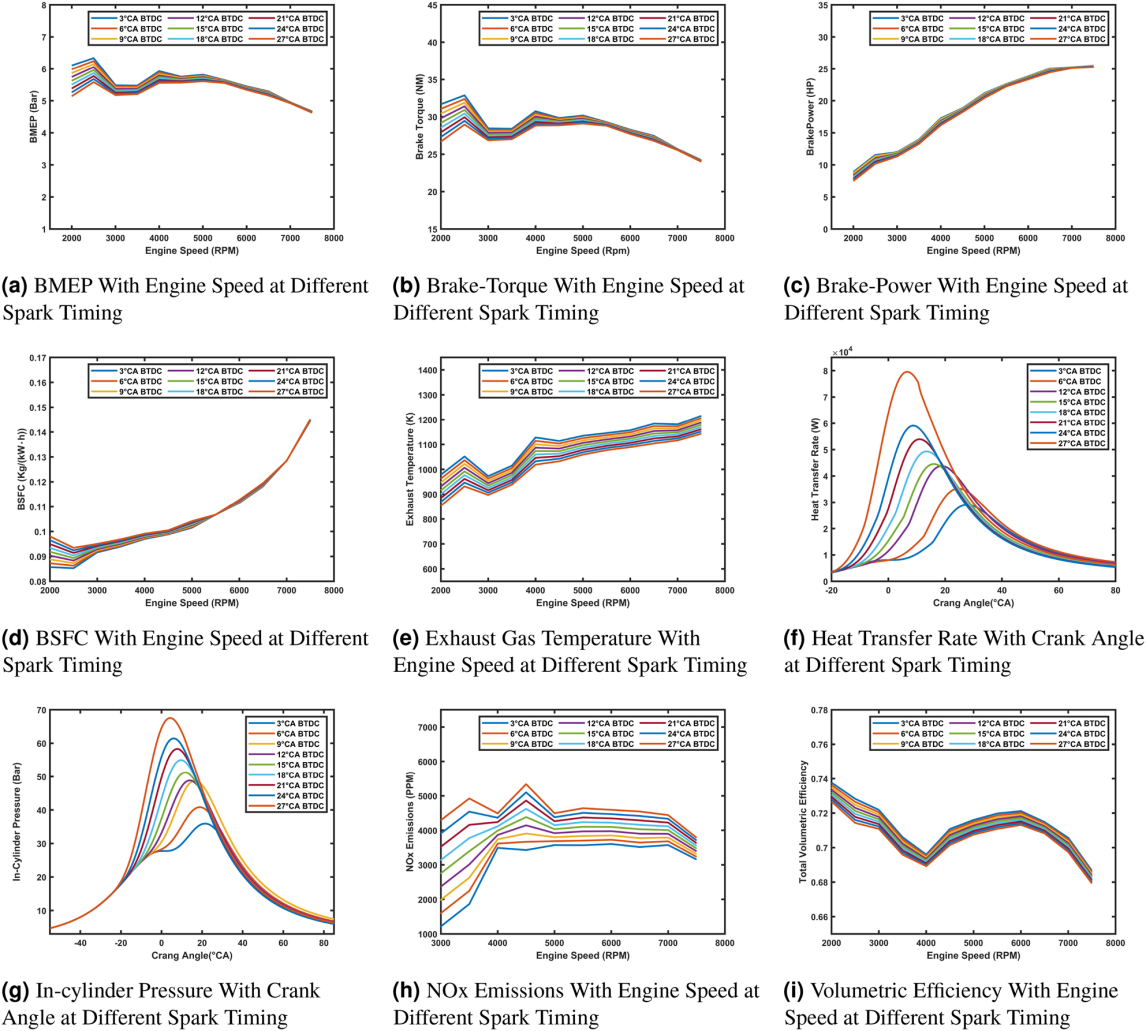
Comparison of engine performance and emissions at different spark timing.

The effect of spark timing on engine performance and emissions is significant across different parameters. BMEP and Brake Torque (Fig. 6a and b) are higher at low engine speeds with retarded spark timing (e.g., 3°CA BTDC), as it optimizes combustion near TDC. At higher speeds, advanced timing (e.g., 24–27° CA BTDC) improves performance by compensating for reduced combustion duration. Brake Power (Fig. 6c) shows minimal variation with spark timing, remaining consistent across RPM.

BSFC (Fig. 6d) is lower with retarded timings at low speeds but increases with advanced timings at high speeds due to incomplete combustion. Exhaust Temperatures (Fig. 6e) are higher with retarded timings, reflecting late combustion, while advanced timings reduce temperatures by enabling more complete combustion. In-Cylinder Pressure and Heat Transfer Rate (Fig. 6g and f) peak earlier and higher with advanced timings, improving efficiency but increasing knocking risk. Retarded timings delay and reduce peak pressures and heat transfer, lowering efficiency but mitigating knocking. NO_x_ Emissions (Fig. 6h) increase with advanced timings due to higher combustion temperatures, while retarded timings reduce NO_x_ formation. Volumetric Efficiency (Fig. 6i) shows minimal sensitivity to spark timing, with retarded timings providing slightly better efficiency at mid-range speeds. These results highlight the importance of balancing spark timing to optimize power, efficiency, and emissions.

### Influence of water injection on engine performance and emissions

Water injection is a critical tool for balancing performance and emission control in hydrogen-powered engines. The HWR significantly influences engine behavior, especially across various engine speeds. This phase evaluates the effects of water injection on Brake Mean Effective Pressure (BMEP), Brake Power, Torque, Brake Specific Fuel Consumption (BSFC), Exhaust Gas Temperature, Heat Transfer Rate, In-Cylinder Pressure, NO_x_ Emissions, and Volumetric Efficiency. By comparing the behavior across different HWRs (1:0.05, 1:0.1, 1:0.2, etc.), the goal is to identify the optimal water injection strategy that balances thermal management, power output, and emissions.

**Fig. 7:**
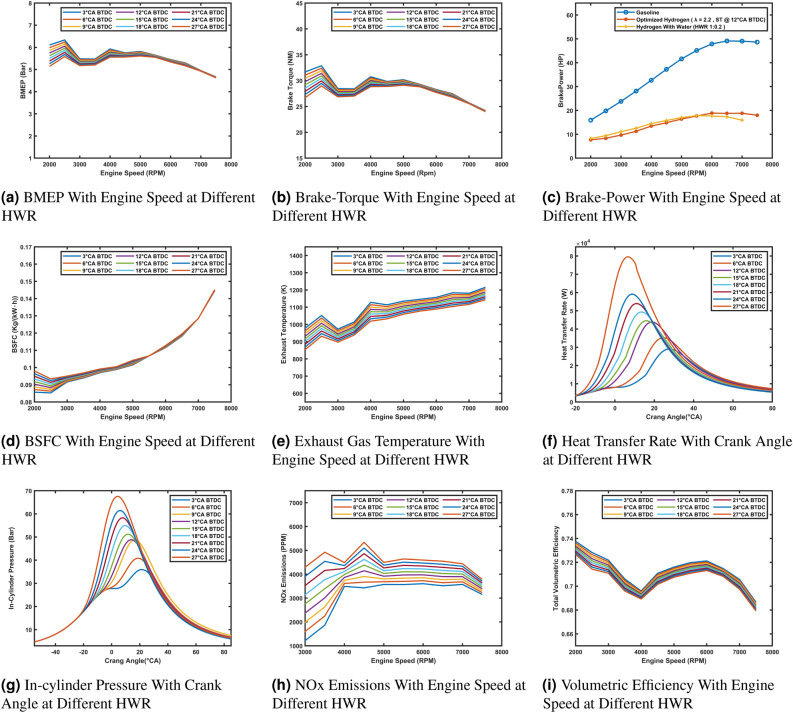
Comparison of engine performance and emissions at different HWR ratios.

Water injection significantly affects engine performance and emissions. BMEP (Fig. 7a) has a higher drop with increasing HWR, at high RPMs, due to the cooling effect reducing peak cylinder pressures. Brake Torque (Fig. 7b) improves at low speeds with higher HWRs but decreases at high speeds due to reduced thermal efficiency. Brake Power (Fig. 7c) shows minimal differences at low RPMs, with higher HWRs slightly improving power above 6000 RPM due to knock suppression.

BSFC (Fig. 7d) decreases with HWR, reflecting improved fuel efficiency, though gains diminish at higher ratios. Exhaust Temperatures (Fig. 7e) drop significantly with increased HWR, reducing thermal stress and NO_x_ formation. In-cylinder pressure (Fig. 7g) and Heat Transfer Rate (Fig. 7f) decrease at higher HWRs, reflecting delayed combustion and reduced heat release. NO_x_ Emissions (Fig. 7h) decline sharply with HWR, particularly above 1:0.2, due to lower combustion temperatures. Volumetric Efficiency (Fig. 7i) improves at low and mid-range RPMs with increased HWR, as cooler intake air increases air density. These results highlight water injection’s potential to enhance efficiency and reduce emissions, though excessive cooling can impact performance at high HWRs.

### Comparative analysis

This section compares the gasoline model, the optimized hydrogen model which operates with Lambda value equal to 2.2, spark timing at 12° CA BTDC, and the hydrogen model with 20% water injection (HWR 1:0.2). The analysis evaluates key performance metrics and combustion characteristics, including Brake Mean Effective Pressure (BMEP), brake torque, brake power, Brake-Specific Fuel Consumption (BSFC), exhaust gas temperature, heat transfer rate, in-cylinder pressure, NO_x_ emissions, and total volumetric efficiency.

**Fig. 8:**
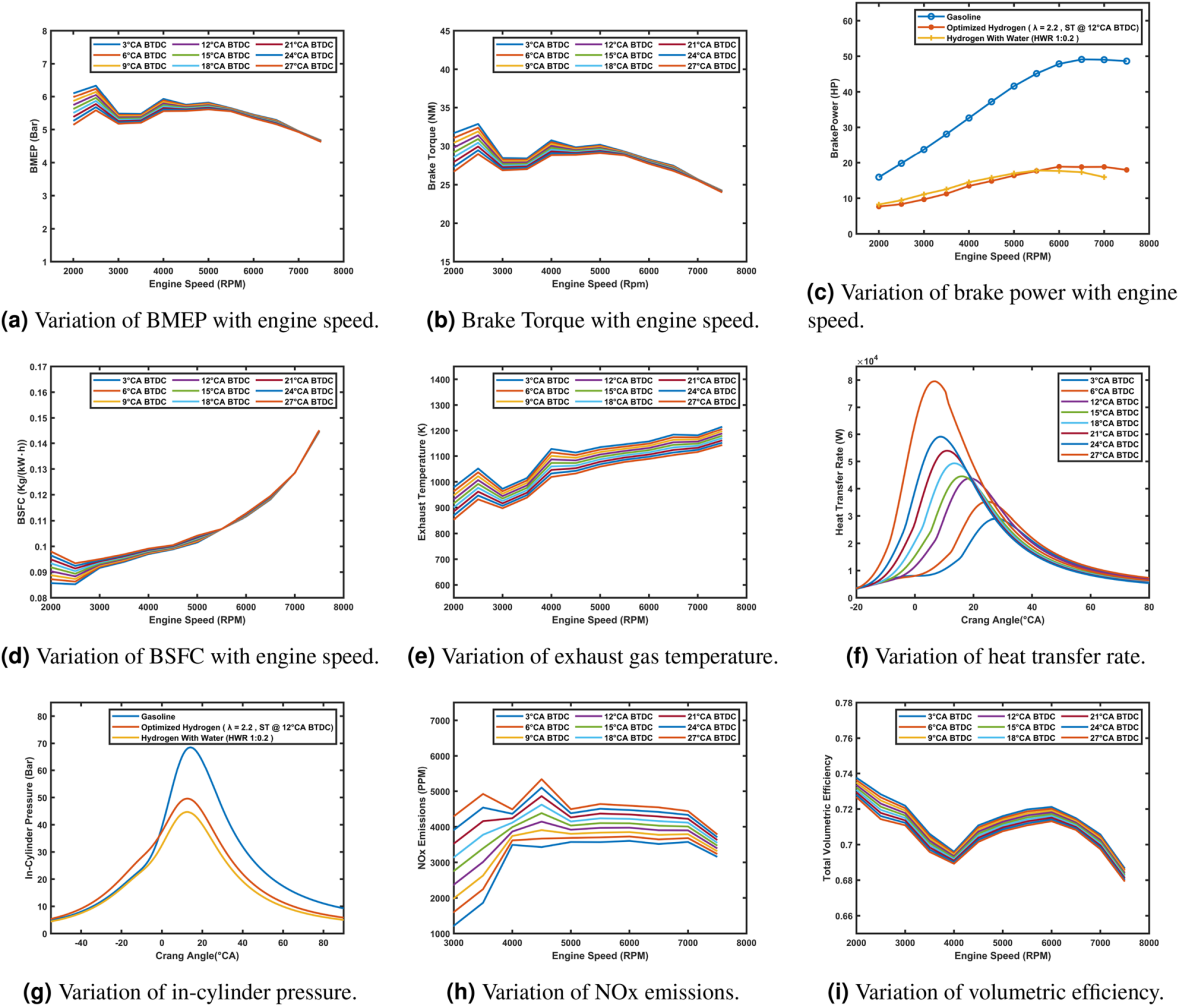
Comparison of gasoline, optimized hydrogen, and hydrogen with water injection models across various performance and emission parameters.

The gasoline model achieves the highest BMEP (Fig. 8a), brake torque (Fig. 8b), brake power (Fig. 8c), and volumetric efficiency (Fig. 8i), benefiting from higher energy density and optimal combustion characteristics. The optimized hydrogen model shows moderate BMEP, torque, and power, with lean-burn efficiency contributing to significantly lower BSFC (Fig. 8d) and reduced NO_x_ emissions (Fig. 8h). The hydrogen model with water injection achieves the lowest BMEP, torque, and power due to the cooling effect of water, which reduces peak pressures and thermal efficiency. However, it performs slightly better at lower RPMs, enhances volumetric efficiency by lowering intake air temperatures, and achieves the lowest NO_x_ emissions and exhaust temperatures (Fig. 8e). Heat transfer rates (Fig. 8f) and in-cylinder pressures (Fig. 8g) are highest for the gasoline model and decrease progressively with hydrogen and water injection, reflecting reduced combustion temperatures and smoother pressure curves that improve durability and reduce knocking tendencies. These findings highlight water injection’s potential for improved efficiency and emissions reduction, albeit with some performance trade-offs.

The comparative analysis highlights the differences between gasoline and hydrogen as fuels. The gasoline model delivers high power and torque but is low in fuel efficiency and emissions. The optimized hydrogen model achieves excellent fuel efficiency and reduced emissions but less performance. The hydrogen model with water injection shows a balance by further reducing emissions, particularly NO_x_, at the cost of slight reductions in power and efficiency. Water injection proves to be an effective strategy for enhancing hydrogen combustion sustainability and making the ability to run a richer hydrogen mixture without worrying about knock.

## Conclusion

This study systematically investigated the effects of air-fuel ratio, spark timing, and water injection on the performance and emissions of a hydrogen-fueled internal combustion engine using 1D simulation. The key conclusions drawn from our comprehensive analysis are as follows:

1. Compared to gasoline operation, hydrogen fueling reduced CO emissions to negligible levels but increased NO_x_ emissions by up to 20% due to higher combustion temperatures, while delivering 63% lower brake-specific fuel consumption.
2. The air-fuel ratio optimization revealed that lean combustion ($\lambda$ = 2.2) provided the best balance between performance and emissions, reducing NO_x_ by 30% while maintaining stable engine operation.
3. Water injection demonstrated significant NO_x_ reduction capabilities, with a hydrogen-to-water ratio of 1:0.2 achieving 50% lower NO_x_ emissions and 5–10% improved volumetric efficiency, though with an 8–12% power output penalty.
4. Spark timing adjustment to 12° CA BTDC proved optimal for minimizing knocking risks while maintaining combustion efficiency, particularly at higher engine speeds.

These findings indicate that hydrogen-fueled engines with proper optimization of operating parameters and water injection strategies can achieve near-zero CO emissions while effectively managing NO_x_ formation. The results provide concrete guidance for implementing hydrogen combustion in practical engine applications, particularly for heavy-duty transportation where emission compliance is critical. The demonstrated 63% improvement in fuel efficiency coupled with effective emission control measures presents a compelling case for hydrogen as a sustainable alternative to conventional fuels. The Table 4 shows the list of abbreviations

**Table 4:** List of abbreviations.

Abbreviation	Description
1D	One-dimensional
AFR	Air-fuel ratio
BMEP	Brake mean effective pressure
BSFC	Brake specific fuel consumption
BTDC	Before top dead center
CA	Crank angle
CI	Compression-ignition
CO	Carbon monoxide
CO_2_	Carbon dioxide
DOE	Design of experiments
EGR	Exhaust gas recirculation
EU	European union
FMEP	Friction mean effective pressure
GHG	Greenhouse gas
H2ICE	Hydrogen-fueled internal combustion engine
HC	Hydrocarbons
HWR	Hydrogen-to-water ratio
ICE	Internal combustion engine
IMEP	Indicated mean effective pressure
NO_x_	Nitrogen oxides
PFI	Port fuel injection
PMEP	Pumping mean effective pressure
RPM	Revolutions per minute
SI	Spark-ignition
THC	Total hydrocarbons
TDC	Top dead center

## Data Availability

The datasets used and/or analyzed during the current study are available from the corresponding author upon reasonable request.
